# Supplementary material for: Rapid functional impairment of natural killer cells following tumor entry limits anti-tumor immunity
Source: Nat Commun. 2024 Jan 24;15:683. doi: 10.1038/s41467-024-44789-z (PMC10808449; doi:10.1038/s41467-024-44789-z)
Supplement: Supplementary file 1 — Supplementary Information [file 41467_2024_44789_MOESM1_ESM.pdf]

Rapid functional impairment of natural killer cells following tumor entry limits anti-tumor immunity

Isaac Dean, Colin Y.C. Lee, Zewen K. Tuong, Zhi Li, Christopher A. Tibbitt, Claire Willis, Fabrina Gaspal, Bethany C. Kennedy, Veronika Matei-Rascu, Rémi Fiancette, Caroline Nordenvall, Ulrik Lindforss, Syed Murtuza Baker, Christian Stockmann, Veronika Sexl, Scott A. Hammond, Simon J. Dovedi, Jenny Mjösberg, Matthew R. Hepworth, Gianluca Carlesso, Menna R. Clatworthy, David R. Withers

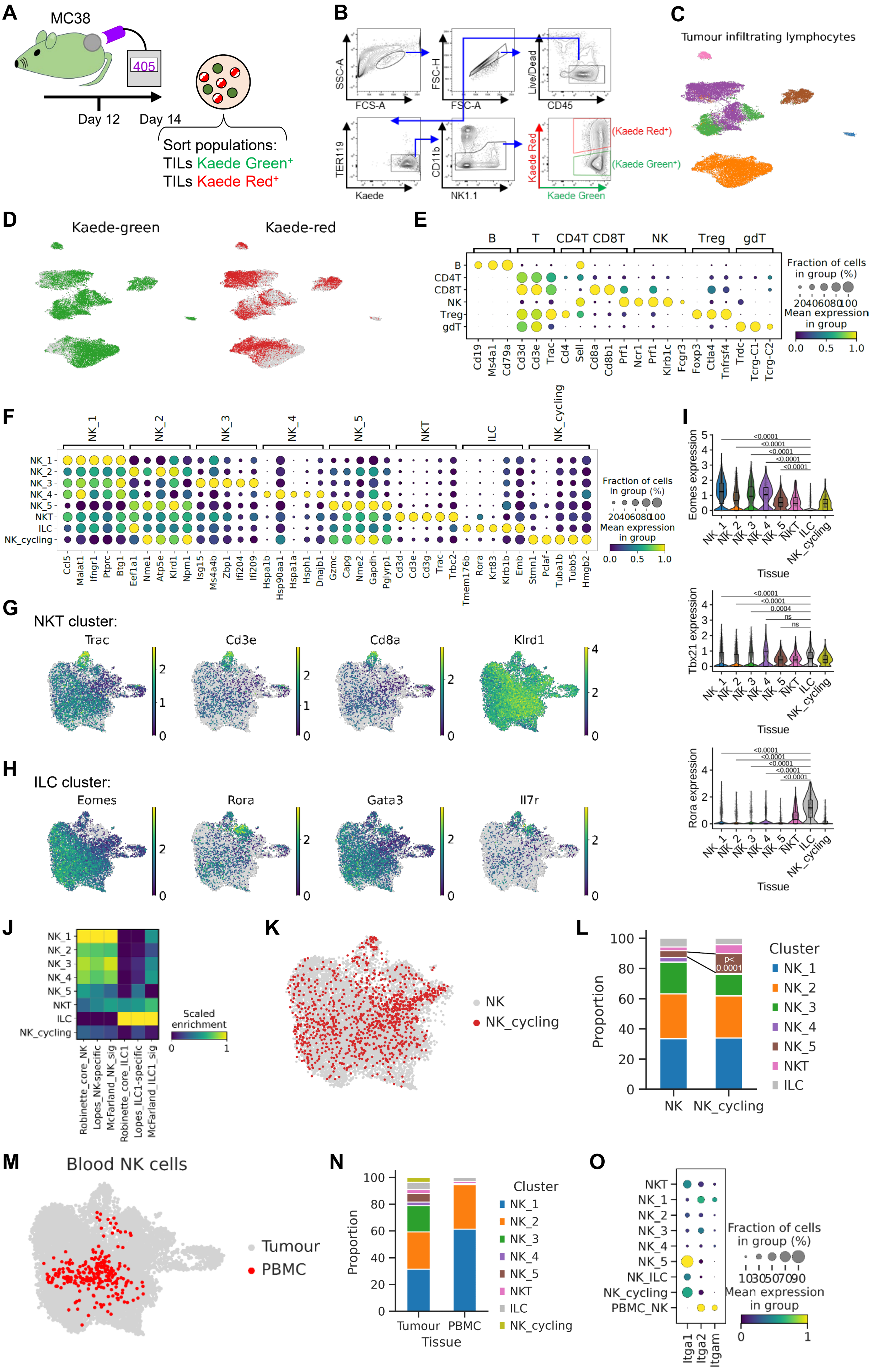

A
MC38
405
Day 12
Day 14
Sort populations:
TILs Kaede Green+
TILs Kaede Red+
B
SSC-A
FCS-A
FSC-H
FSC-A
Live/Dead
CD45
TER119
Kaede
CD11b
NK1.1
Kaede Red
Kaede Green
(Kaede Red+)
(Kaede Green+)
C
Tumour infiltrating lymphocytes
B
CD4T
CD8T
NK
Treg
gdT
D
Kaede-green
Kaede-red
E
F
G
NKT cluster:
Trac
Cd3e
Cd8a
Klrd1
H
ILC cluster:
Eomes
Rora
Gata3
Il7r
I
Eomes expression
Tbx21 expression
Rora expression
Tissue
J
Scaled enrichment
Robinette_core_NK
Lopes_NK-specific
McFarland_NK_sig
Robinette_core_ILC1
Lopes_ILC1-specific
McFarland_ILC1_sig
K
NK
NK_cycling
L
Proportion
Cluster
p< 0.0001
M
Blood NK cells
Tumour
PBMC
N
Proportion
Tissue
Cluster
O
Fraction of cells in group (%)
Mean expression in group
Itga1
Itga2
Itgam

Supplementary Fig. 1. Single cell RNA-sequencing of Tumor Infiltrating Lymphocytes 48 hrs after photoconversion.

(A) Cartoon summarizing experimental plan to isolate photo-labelled TILs from MC38 tumors for analysis by scRNA-seq. (B) Gating strategy used to isolate TILs and ensure appropriate capture of NK cells. (C) UMAP showing immune cell clusters within the total TIL compartment. (D) UMAP showing the distribution of Kaede Green+ and Kaede Red+ cells amongst the immune cell clusters (E) Dot plots showing canonical marker expression used to initially define major cell clusters. (F) Dot plots showing differentially expressed genes defining the 8 clusters comprising the initial main 'NK cell' cluster in 'C'. (G) UMAPs summarizing expression of key genes used to define the NKT cluster. (H) UMAPS summarizing expression of key genes used to define the ILC cluster. (I) Violin plots showing expression of *Tbx21*, *Eomes*, and *Rora* across the clusters. (J) Enrichment of ILC1 and NK signatures from Robinette et al. 2015[37], McFarland et al., 2021[21], Lopes et al. 2022[23] across the NK/ILC/NKT clusters. (K) UMAP showing re-integration and label transfer of Mki67+ cells.reintegration of *Mki67*+ cells following regression of cell cycle genes. (L) Proportion of cells assigned to NK-cycling cluster represented in the original NK/ILC/NKT clusters, *note significantly increased proportion of cells in NK_5,* assessed by Chi-square test. (M) UMAP showing NK cells from murine PBMCs integrated with the NK clusters. (N) Proportion of PBMC NK cells mapped to tumor NK cell clusters by integration and label transfer. (O) Expression of *Itga1*, *Itga2* and *Itgam* in tumor NK cells and PBMCs. Statistical significance was determined by (I) two-sided Wilcoxon rank-sum test with Benjamini-Hochberg multiple-testing correction, data are shown as box (median; box, 25th percentile and 75th percentile; whiskers, 1.5*inter-quartile range) and violin plots; (L) Chi-squared test for over-representation of NK_5.

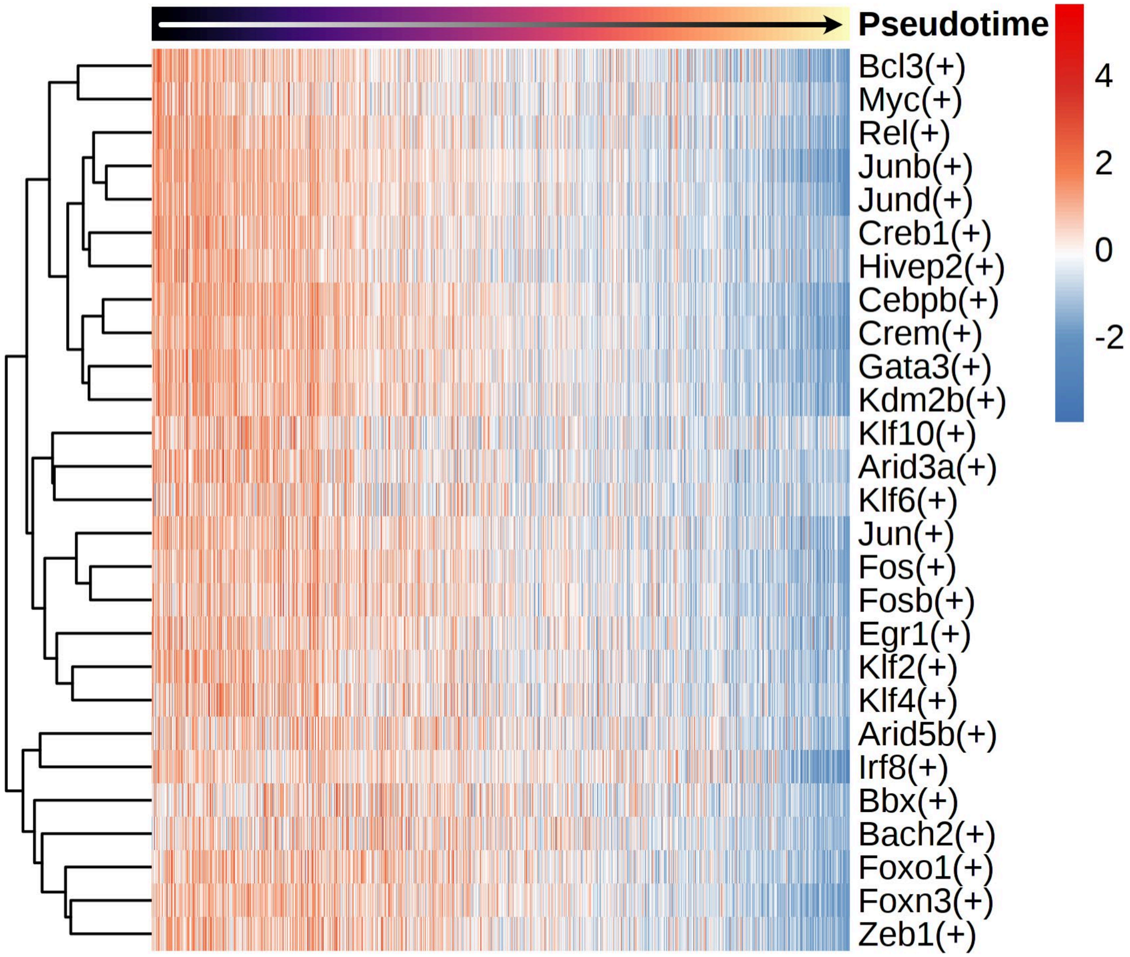

Supplementary Fig. 2. Changes in transcription factor regulon expression over pseudotime.
SCENIC was used to predict/analyse gene regulatory networks and transcription factor activity in scRNA-seq of intratumoral NK cells over pseudotime.

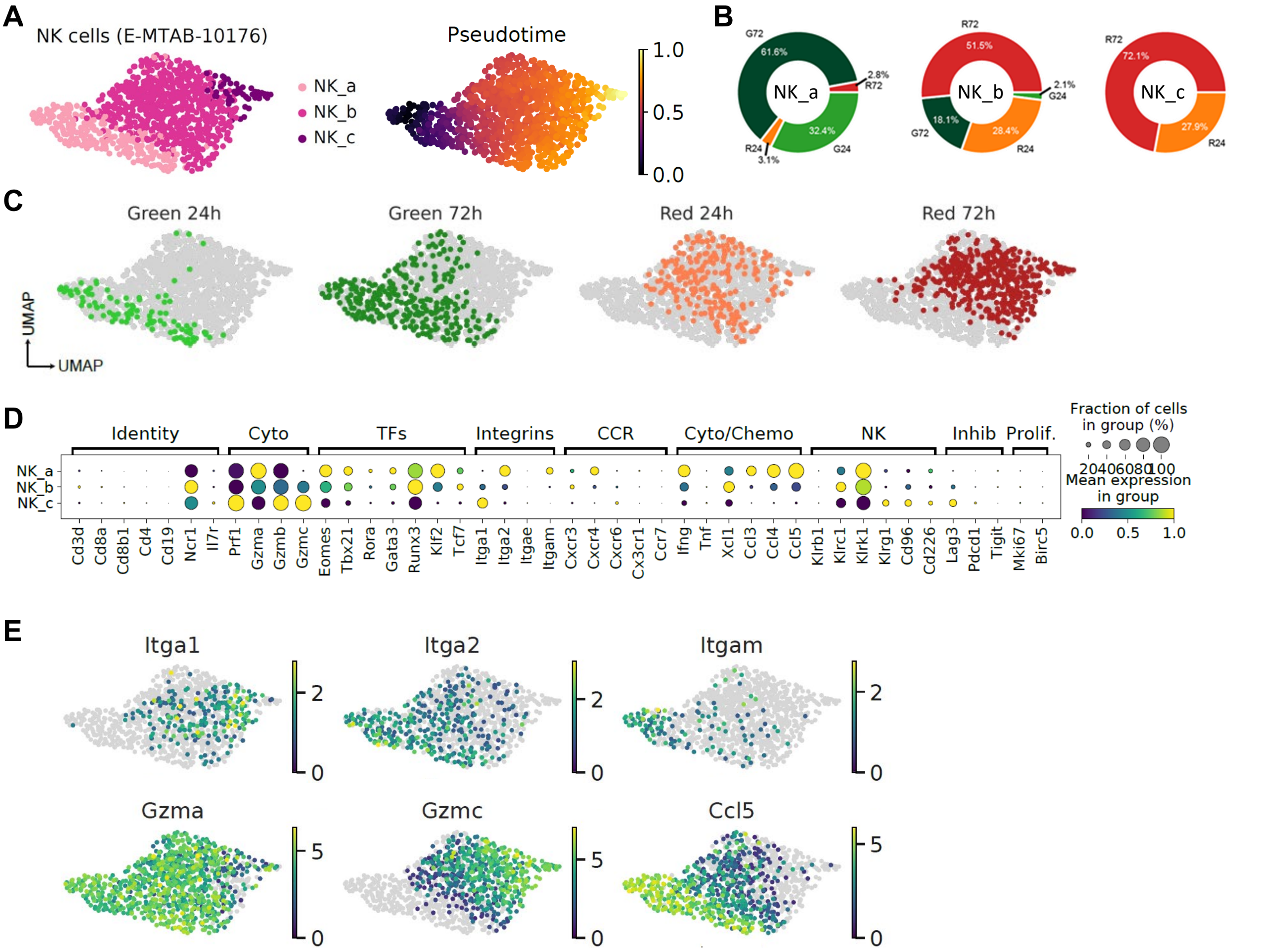

Supplementary Fig. 3. Tracking transcriptomic changes in tumor infiltrating NK cells over time. To further investigate how NK cells change over time within the TME, scRNA-seq data generated from FACS-isolated TILs at two time points post photoconversion of MC38 tumors[34] was reanalyzed (data set E-MTAB-10176). This data set contained 4 samples, comprised of Kaede Green+ and Kaede Red+ TILs each at 24 and 72 hrs post photoconversion. (A) UMAPs showing the three NK clusters identified in the data set (NK_a, NK_b, NK_c) and the pseudotime trajectory rooted in NK_a. (B) Proportion of NK cells from the different samples in each cluster, with NK_a almost entirely dominated by Kaede Green+ cells and NK_c comprised of only Kaede Red+ cells. (C) UMAPs showing the distribution of cells in each sample across the three clusters. (D) Dot plots showing expression of selected genes used to further characterize the clusters. Note, this is the same genes assessed in Fig. 1D. (E) UMAPs showing expression of selected genes across the NK clusters, highlighting differential expression of integrins, granzymes and *Ccl5*.

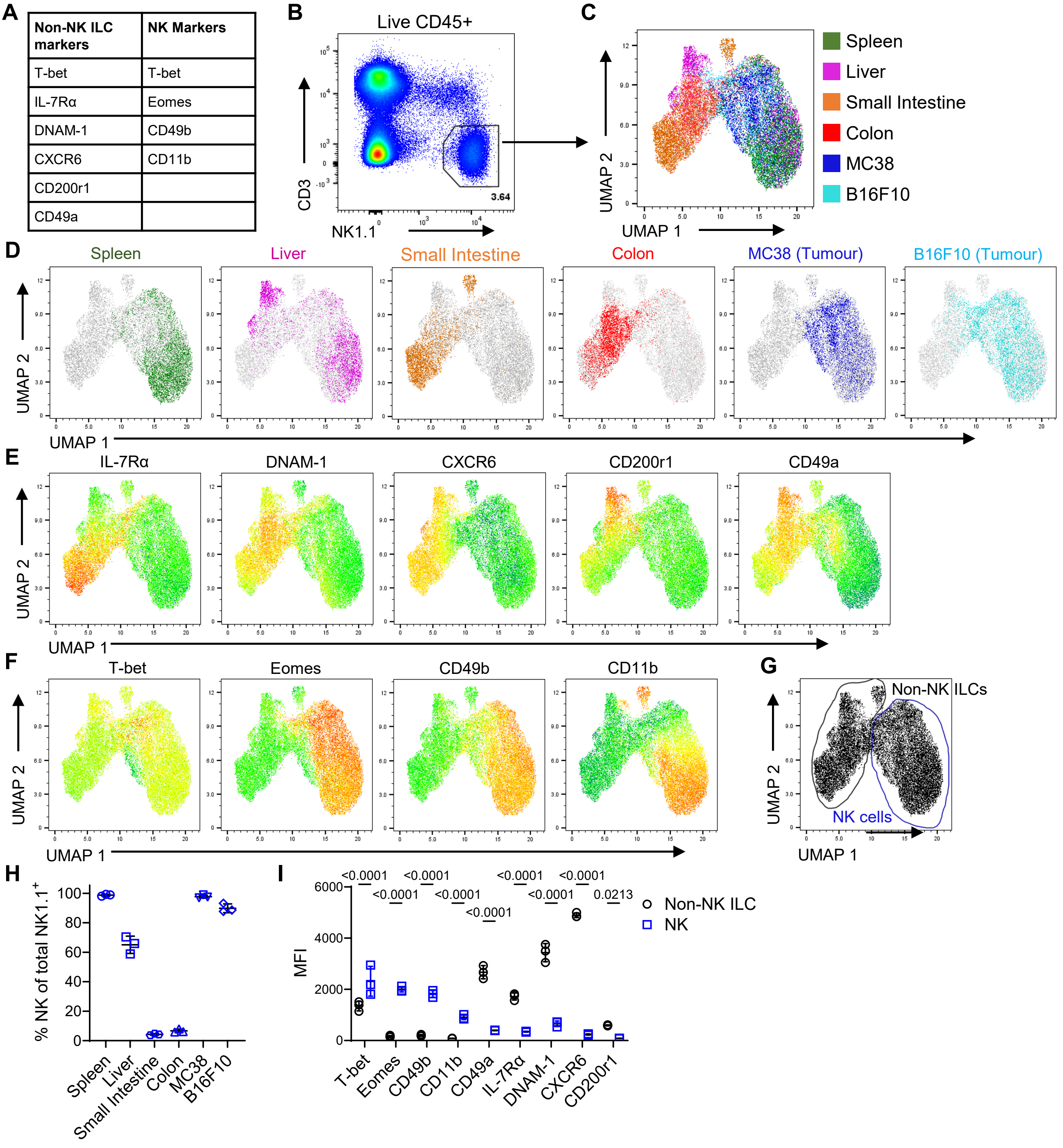

Supplementary Fig. 4. The CD3- NK1.1+ compartment of tumors is phenotypically distinct to that of other tissues.

To compare the phenotype of NK cells within tumors to the NK and ILC1 cells found in healthy tissue, flow cytometry was used to assess cells isolated from MC38 and B16-F10 tumors as well as spleen, liver, small intestine, and colon. (A) Table showing the ILC and NK cell 'markers' analyzed. (B) Flow cytometry plot showing gating on CD3- NK1.1+ cells (amongst live CD45+ cells). (C) UMAP showing 4,500 $CD3^-$ $Nk1.1^+$ cells from each tissue in non-tumor bearing mice (n=3), and from MC38 and B16F10 tumors (n=3). (D) UMAPS showing the distribution of cells in each tissue. (E) UMAPs showing expression of proteins typically associated with ILCs rather than NK cells (IL-7Rα, DNAM-1, CXCR6, CD200r1, CD49a). (F) UMAPs showing expression of proteins typically associated with NK cells (T-bet, Eomes, CD49b, CD11b). (G) UMAP summarizing the distribution of NK cells versus non-NK ILCs. (H) The proportion of cells within the CD3- NK1.1+ gate that are NK cells within each tissue, defined in 'G'. (I) Graph showing MFI of selected proteins in the non-NK ILC and NK regions of the UMAP defined in 'G'. Statistical significance was determined by two-way ANOVA with Šidák's multiple comparisons test. Data are presented showing all individual data points as well as the mean value +/- SD.

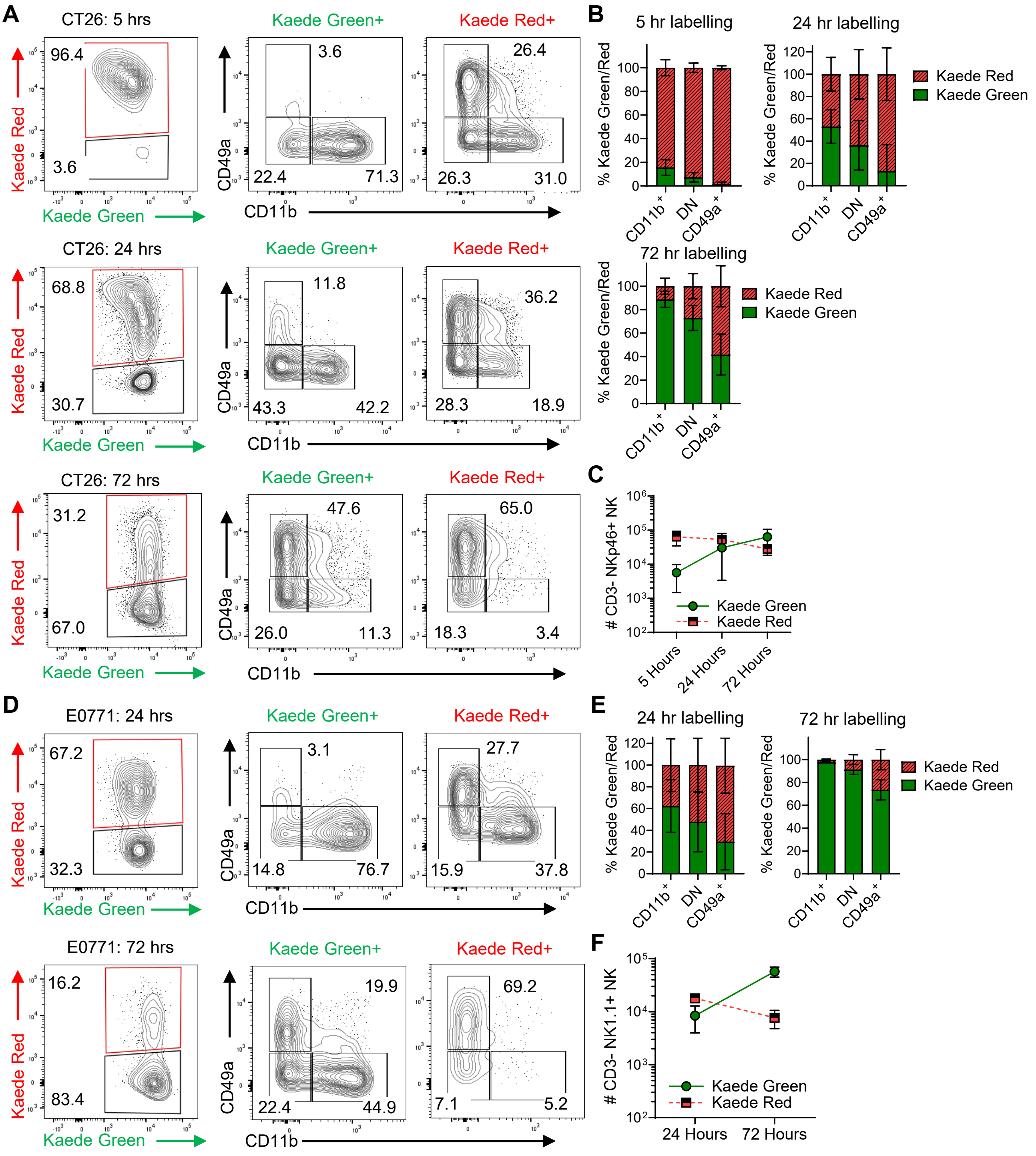

Supplementary Fig. 5. Changes in NK cell integrin expression over time observed in multiple tumor models.

The changes in NK cell integrin expression over time in the TME observed in MC38 tumors were further assessed in CT26 and E0771 tumors, grafted subcutaneously into BALB/c Kaede and into the mammary fat pad of C57BL/6 Kaede mice respectively. (A) Expression of CD49a versus CD11b by Kaede Green+ and Kaede Red+ NK cells (CD3- NKp46+) isolated from CT26 tumors at 5 (n=5), 24 (n=5), and 72 (n=6) hrs post photoconversion. Data shown for 1 independent repeat at 5 hrs and 24 hrs, and 2 pooled independent repeats at 72 hrs post-labeling. (B) The proportion of Kaede Green/Red for each NK cell subset at each time point post photoconversion. (C) Total number of Kaede Green+ and Kaede Red+ NK cells at each time point post photoconversion. (D) Expression of CD49a versus CD11b by Kaede Green+ and Kaede Red+ NK cells (CD3- NK1.1+) isolated from E0771 tumors at 24 (n=9) and 72 (n=4) hrs post photoconversion. Data shown for 2 pooled independent repeats at 24 hrs, and 1 independent repeat at 72 hrs post-labeling. (E) The proportion of Kaede Green/Red for each NK cell subset at each time point post photoconversion. (F) Total number of Kaede Green+ and Kaede Red+ NK cells at each time point post photoconversion. Data are presented showing all individual data points as well as the mean value +/- SD.

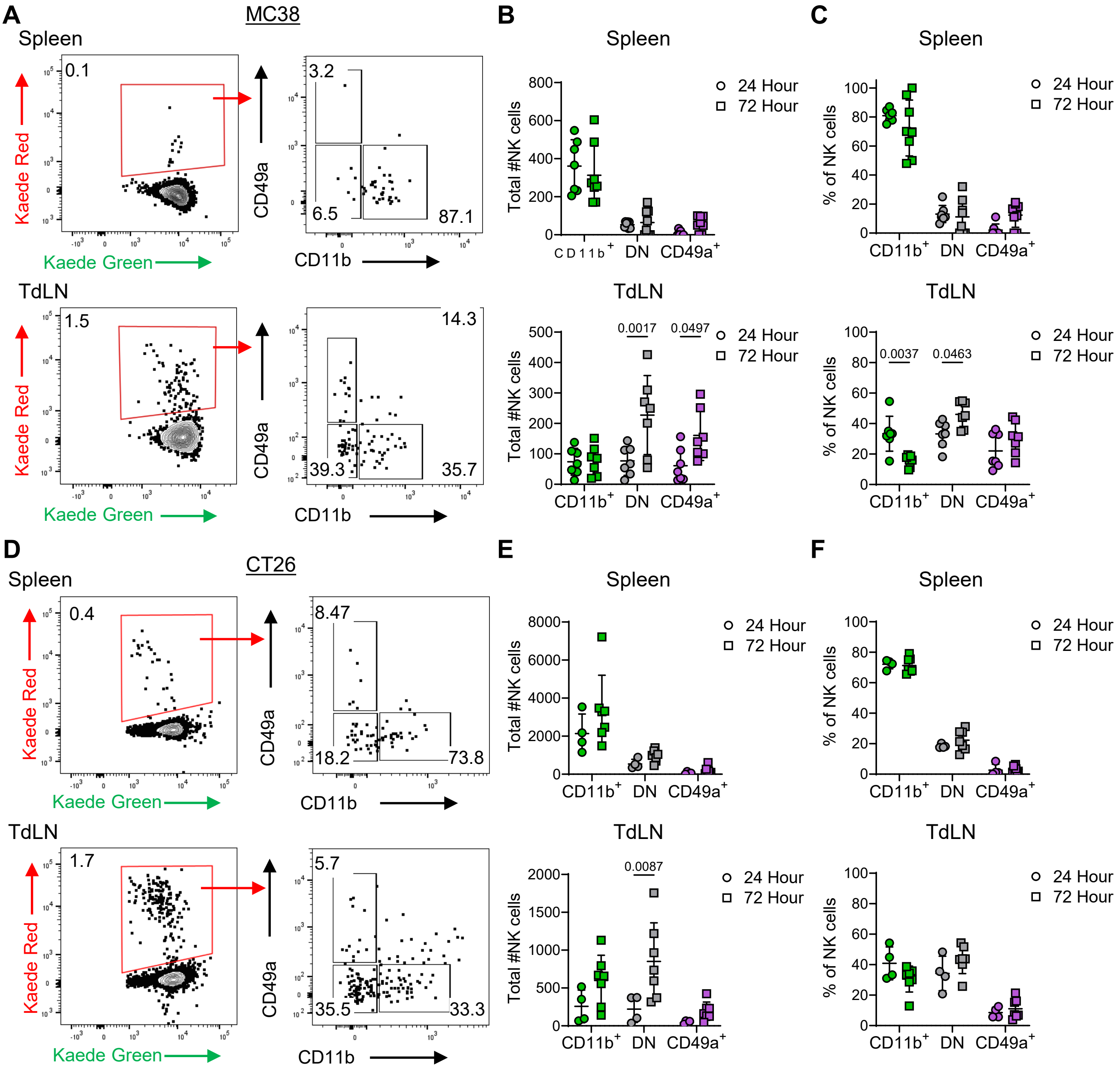

Supplementary Fig. 6. Limited NK cell egress from tumors.
To investigate the egress of NK cells from tumors, MC38 and CT26 tumors were photoconverted and the Kaede Red+ cells within the draining (inguinal) lymph node and spleen assessed at 24 and 72 hrs post photoconversion. (A) Identification of Kaede Red+ NK cells (CD3- NK1.1+) in the spleen and dLN of mice bearing MC38 tumors, with CD49a versus CD11b expression used to assess NK cell phenotype. (B) Total number on NK cells within the spleen and dLN at 24 (n=7) and 72 (n=8) hrs post photoconversion, showing NK cell subsets defined by CD49a versus CD11b expression. Data pooled from 2 independent experiments. (C) Proportion of NK cells within the spleen and dLN at 24 and 72 hrs post photoconversion in each NK cell subset defined by CD49a versus CD11b expression. (D) Identification of Kaede Red+ NK cells (CD3- NKp46+) in the spleen and dLN of mice bearing CT26 tumors, with CD49a versus CD11b expression used to assess NK cell phenotype. (E) Total number on NK cells within the spleen and dLN at 24 (n=4) and 72 (n=7) hrs post photoconversion, showing NK cell subsets defined by CD49a versus CD11b expression. Data from 1 independent experiment. (F) Proportion of NK cells within the spleen and dLN at 24 and 72 hrs post photoconversion in each NK cell subset defined by CD49a versus CD11b expression. Statistical significance was determined by two-way ANOVA with Šidák's multiple comparisons test. Data are presented showing all individual data points as well as the mean value +/- SD.

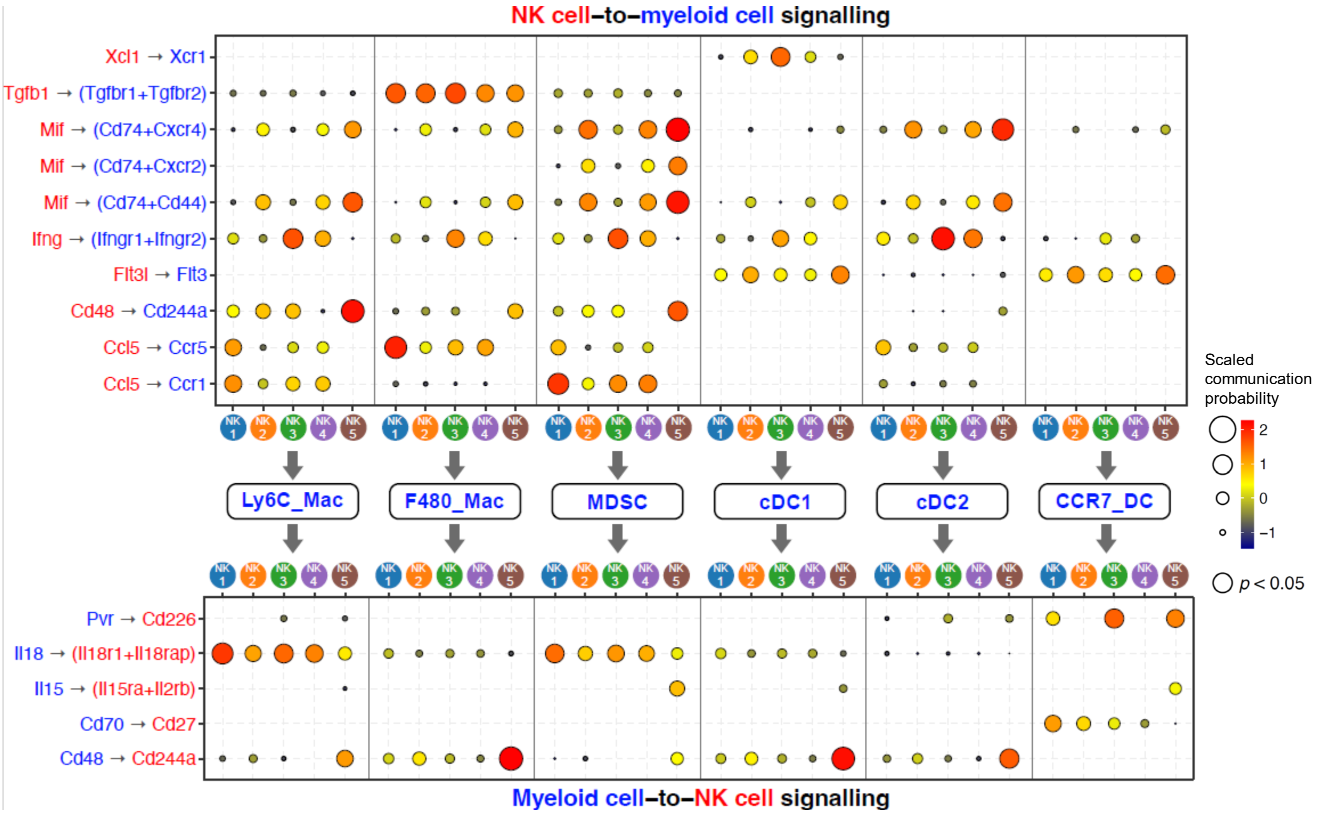

Supplementary Fig. 7. Analysis of NK:myeloid cell-cell communications using CellChat.
Cell-cell communication analysis (CellChat) showing selected predicted ligand-receptor interactions between scRNA-seq of NK cells and myeloid cell types. Only significant interactions are shown.

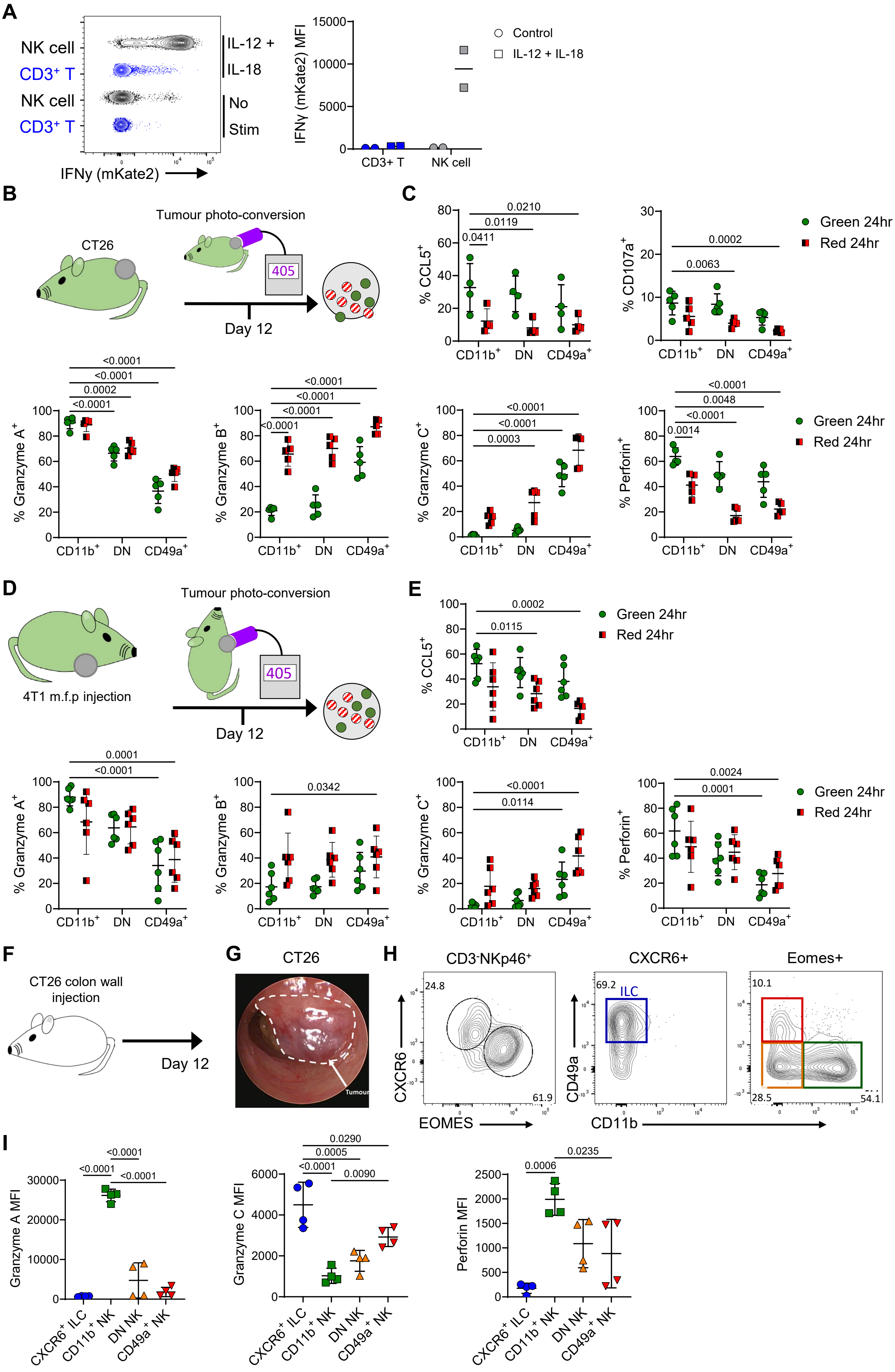
A
NK cell
CD3+ T
NK cell
CD3+ T
IL-12 +
IL-18
No
Stim
IFNγ (mKate2)
IFNγ (mKate2) MFI
Control
IL-12 + IL-18
CD3+ T
NK cell
B
CT26
Tumour photo-conversion
405
Day 12
C
% CCL5+
% CD107a+
Green 24hr
Red 24hr
CD11b+
DN
CD49a+
% Granzyme A+
% Granzyme B+
% Granzyme C+
% Perforin+
D
4T1 m.f.p injection
Tumour photo-conversion
405
Day 12
E
F
CT26 colon wall
injection
Day 12
G
CT26
Tumour
H
CD3-NKp46+
CXCR6+
Eomes+
CXCR6
EOMES
CD49a
CD11b
ILC
I
Granzyme A MFI
Granzyme C MFI
Perforin MFI
CXCR6+ ILC
CD11b+ NK
DN NK
CD49a+ NK

Supplementary Fig. 8. Confirmation of changes in NK cell function over time in the TME across multiple pre-clinical tumor models.

(A) Representative expression of mKate2 as a read-out for *Ifng* expression by NK cells versus T cells after *ex vivo* culture of splenocytes ± IL-12 and IL-18 (n=2). (B) Cartoon showing experimental set up with CT26 tumors grafted subcutaneously on the flank of BALB/c Kaede mice and photoconverted 12 days later. (C) Proportion of NK cells producing CCL5, CD107a, Granzyme A, Granzyme B, Granzyme C, and Perforin after *ex vivo* stimulation at 24 hrs post photoconversion. Data representative of 2 independent experiments, CCL5 n=4, all other parameters n=5. (D) Cartoon showing experimental set up with 4T1 tumors grafted into the mammary fat pad (m.f.p) of BALB/c Kaede mice and photoconverted 12 days later. (E) Proportion of NK cells producing CCL5, Granzyme A, Granzyme B, Granzyme C, and Perforin after *ex vivo* stimulation at 24 hrs post photoconversion. Data was pooled from 2 independent experiments, n=6 at each time point. (F) Cartoon showing experimental set up with CT26 tumors grafted into the colon wall of C57BL/6 Kaede mice via endoscopy. (G) Endoscopic photo of the CT26 tumor growing within the colon 12 days after injection. (H) Flow cytometry plots showing identification of CXCR6+ ILC and Eomes+ NK cells within the CD3- NKp46+ population within the tumor. Further analysis of CD49a versus CD11b revealed the presence of three subsets within the Eomes+ NK cells, consistent with analysis of CT26 tumors grown subcutaneously. (I) MFI of Granzyme A, Granzyme C and Perforin expression. Data from 1 independent experiment, n=4. Statistical significance was determined by (C, E) two-way ANOVA with Šidák's multiple comparisons test; (I) one-way ANOVA with Tukey's multiple comparison test comparing all means. Data are presented showing all individual data points as well as the mean value +/- SD throughout, except in (A) where only the mean is shown.

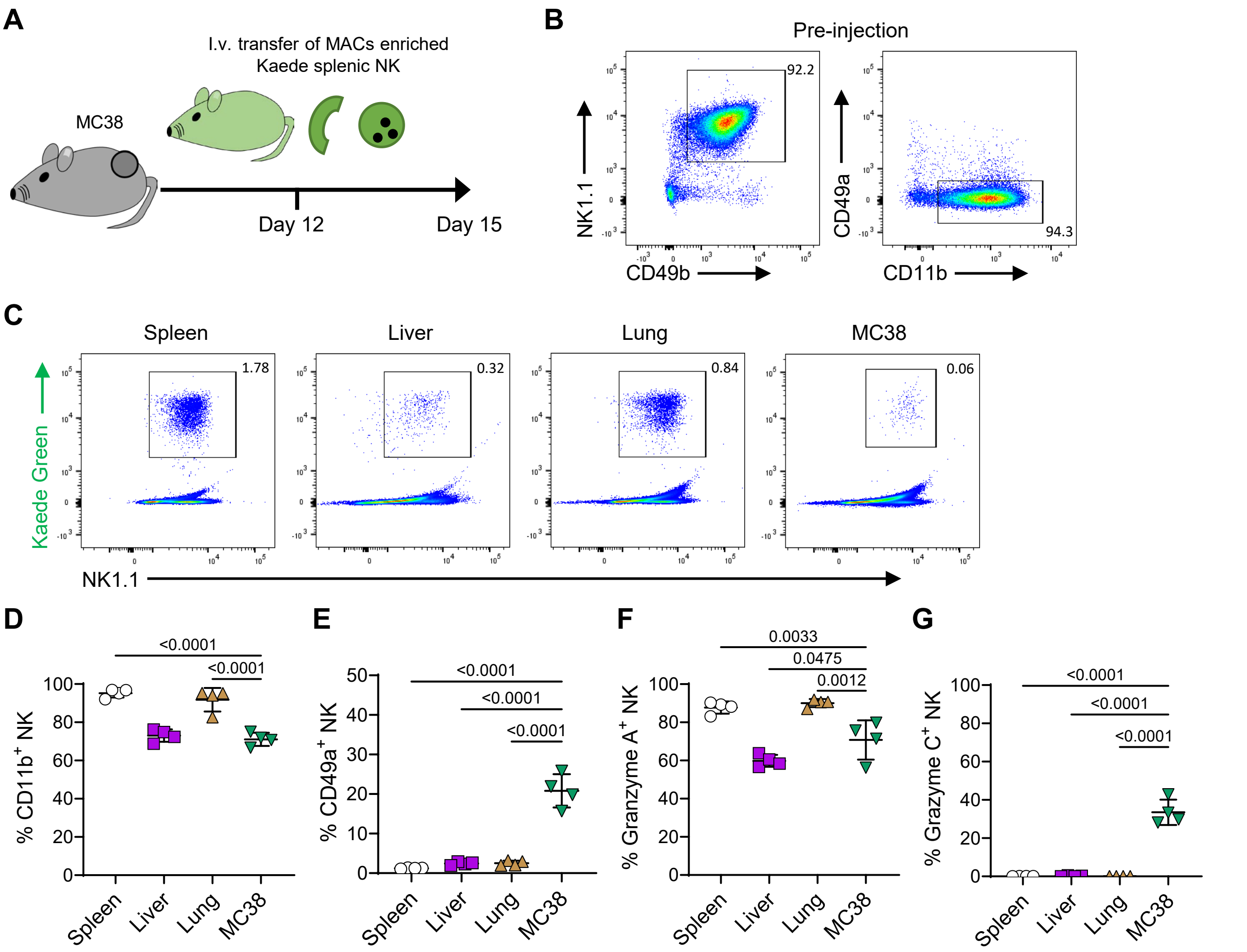

Supplementary Fig. 9. Transferred splenic NK cells upregulate CD49a and Granzyme C expression after entry into MC38 tumors but not healthy tissues.

Approximately 250,000 MACS-enriched NK cells isolated from the spleens of C57BL/6 Kaede mice and injected intravenously into wild-type C57BL/6 mice bearing MC38 tumors (n=4). Three days after transfer, mice were culled and cells isolated from spleen, liver, lung, and MC38 tumors analyzed after *ex vivo* restimulation. (A) Cartoon summarizing the experimental design. (B) Flow cytometry plots showing efficiency of NK cell MACS-enrichment. (C) Flow cytometry plots showing identification of transferred Kaede Green+ NK cells in different tissues. Proportion of transferred NK cells expressing CD11b (D), CD49a (E), Granzyme A (F) and Granzyme C (G) in each tissue. Data representative of 2 independent experiments. Statistical significance was determined by one-way ANOVA with Dunnett's multiple comparison test comparing means to MC38. Data are presented showing all individual data points as well as the mean value +/- SD.

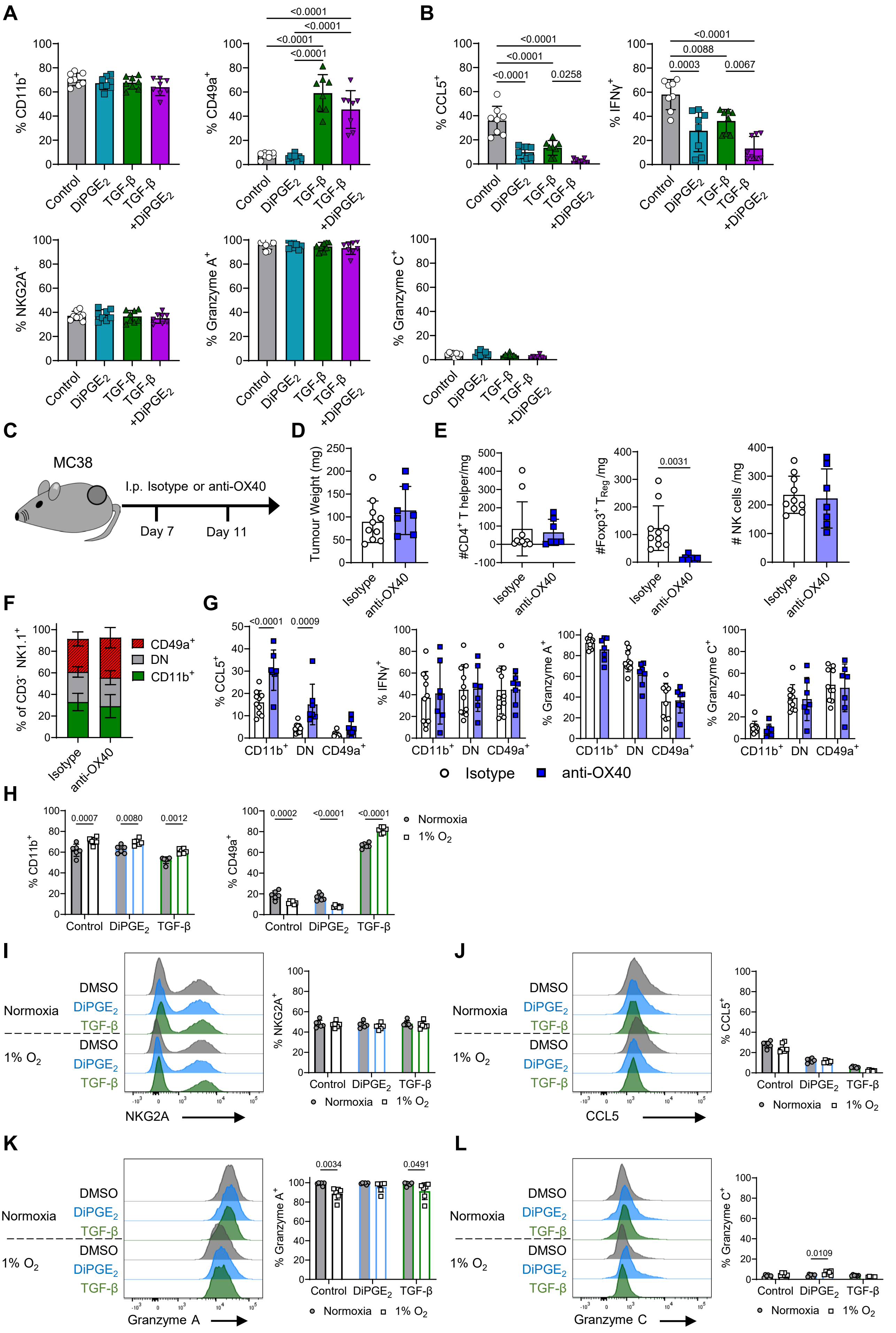
A
B
C
D
E
F
G
H
I
J
K
L
% CD11b+
% CD49a+
% CCL5+
% IFNγ+
% NKG2A+
% Granzyme A+
% Granzyme C+
Control
DiPGE2
TGF-β
TGF-β +DiPGE2
<0.0001
0.0088
0.0003
0.0067
0.0258
MC38
I.p. Isotype or anti-OX40
Day 7
Day 11
Tumour Weight (mg)
#CD4+ T helper/mg
#Foxp3+ TReg/mg
NK cells /mg
0.0031
Isotype
anti-OX40
% of CD3- NK1.1+
CD49a+
DN
CD11b+
0.0009
0.0007
0.0080
0.0012
0.0002
Normoxia
1% O2
DMSO
NKG2A
CCL5
Granzyme A
Granzyme C
0.0034
0.0491
0.0109

Supplementary Fig. 10. Investigating the contributions of TGF-β and PGE_2 *in vitro*, and Tregs *in vivo, in* suppressing NK cell effector functions.

TGFβ, PGE2, and hypoxia were investigated *in vitro* and *in vivo* as potential mechanisms promoting NK cell differentiation to the CD49a expression tumor retained state. Splenic NK cells isolated and then cultured with IL-2/IL-15 further supplemented with TGF-β and/or PGE_2 for 48 hrs, restimulated *ex vivo* and analyzed by flow cytometry. (A) Bar chart showing proportion of CD11b+ and CD49a+ NK cells after 48 hrs in culture in RPMI with IL-2/IL-15 further supplemented with TGF-β and/or DiPGE2. (B) Bar charts showing proportion of CCL5, IFNγ, NKG2A, Granzyme A, and Granzyme C expressing NK cells. (C) Cartoon summarizing experimental design for depleting Tregs in MC38 tumors to impede TGFβ production *in vivo*. WT C57BL/6 mice treated with PBS (n=11) or anti-OX40 antibodies (OX-86, mIgG2A, n=7) on D7 and D11, then analyzed 2 days later. (D) Tumor weight. (E) Enumeration of different TIL populations, showing CD4+ FoxP3- T cells, FOXP3+ Tregs and NK cells. (F) Proportion of NK cells in CD11b+ CD49a-, CD11b- CD49a- and CD11b- CD49a+ subsets. (G) Proportion of NK cells producing CCL5, IFNγ, Granzyme A, Granzyme C after *ex vivo* restimulation. (H) Splenic NK cells were isolated and cultured with +IL-2/IL-15, in addition to TGF-b or DiPGE2 in either a 1% O2 hypoxic chamber or normal incubator. After 2 days cells were then restimulated with PMA and Ionomycin and analyzed. Bar chart comparing proportion of CD11b+ NK cells, and CD49a+ cells. Representative histograms alongside proportion of NK cells positive for (I) NKG2A, (J) CCL5, (K) Granzyme A, or (L) Granzyme C. Statistical significance was determined by (A, B) one-way ANOVA with Tukey's multiple comparison test; (C, G, L) two-way ANNOVA with Šidák's multiple comparison test; (I, J) unpaired t-tests. Data are presented showing all individual data points as well as the mean value +/- SD.

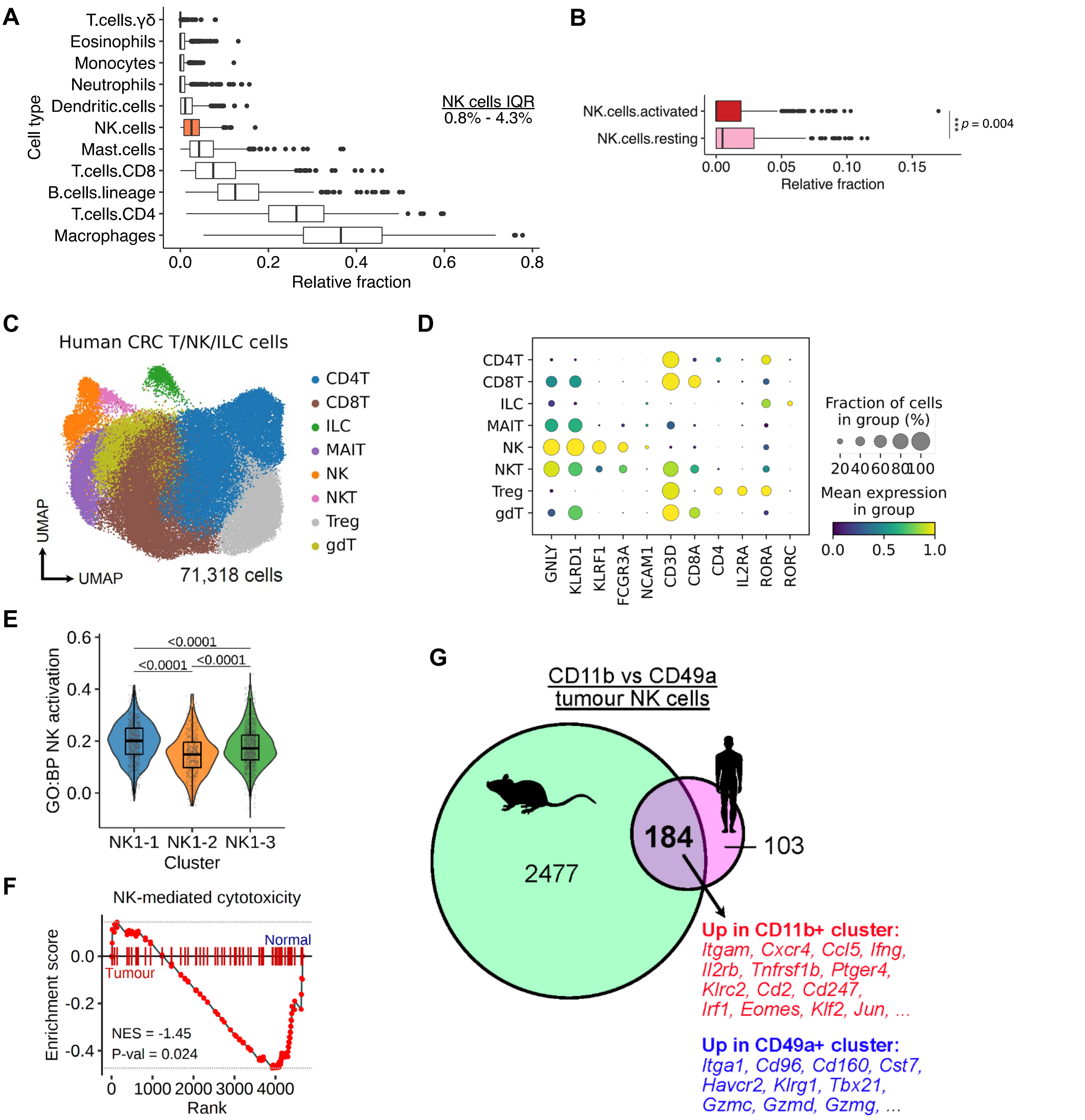

Supplementary Fig. 11. Further analysis of human CRC data.
(A) CIBERSORTx deconvolution of immune cells from bulk transcriptomes of 521 human colorectal cancers, from TCGA. (B) Further subdivision of NK cells from deconvolution results in (A). (C) UMAP of 71,318 T, NK or ILC cells from scRNA-seq of 62 human CRC samples, from GSE178341, and (D) canonical marker gene expression. (E) Gene set enrichment for GO:BP Natural killer cell activation between NK1 clusters. (F) Gene set enrichment analysis (GSEA) of KEGG Natural killer cell-mediated cytotoxicity between scRNA-seq of NK cells from tumor and normal adjacent tissue, in human CRC. (G) Venn diagram showing DEGs between *Itgam*-expressing and *Itga1*-expressing tumor NK cells in murine MC38 tumors (Fig. 1A) and human CRC (Fig. 5A). Statistical significance was determined by two-sided Wilcoxon rank-sum test. In (B) (B) Data are shown as box plots (median; box, 25th percentile and 75th percentile; whiskers, 1.5*inter-quartile range; dots, outliers). In (E) Data are shown as box (median; box, 25th percentile and 75th percentile; whiskers, 1.5*inter-quartile range) and violin plots.

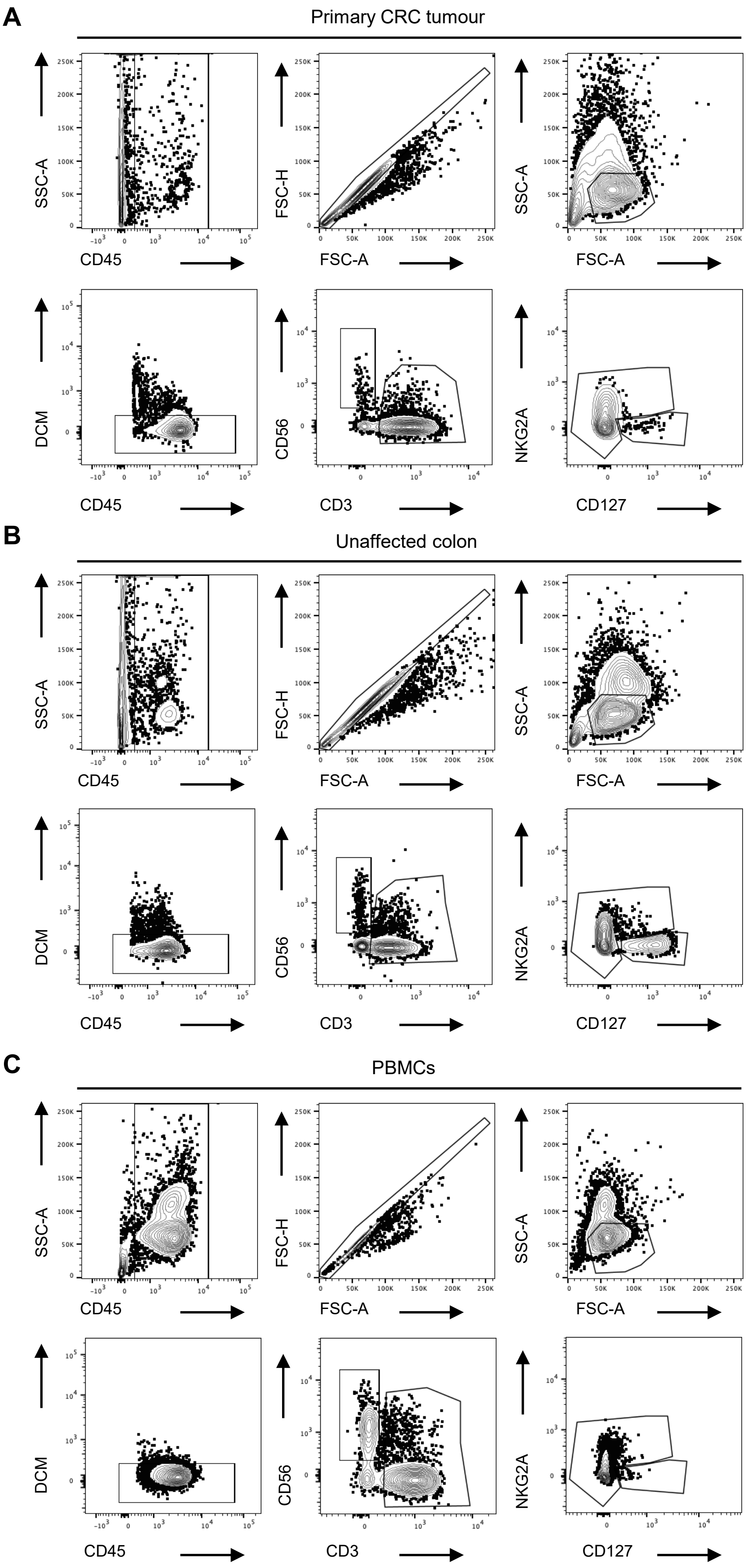

Supplementary Fig. 12. Gating strategies for identification of human NK cells.
Representative plots showing gating strategy for NK cell subsets from (A) primary CRC tumor, (B) unaffected colon tissue, and (C) blood.

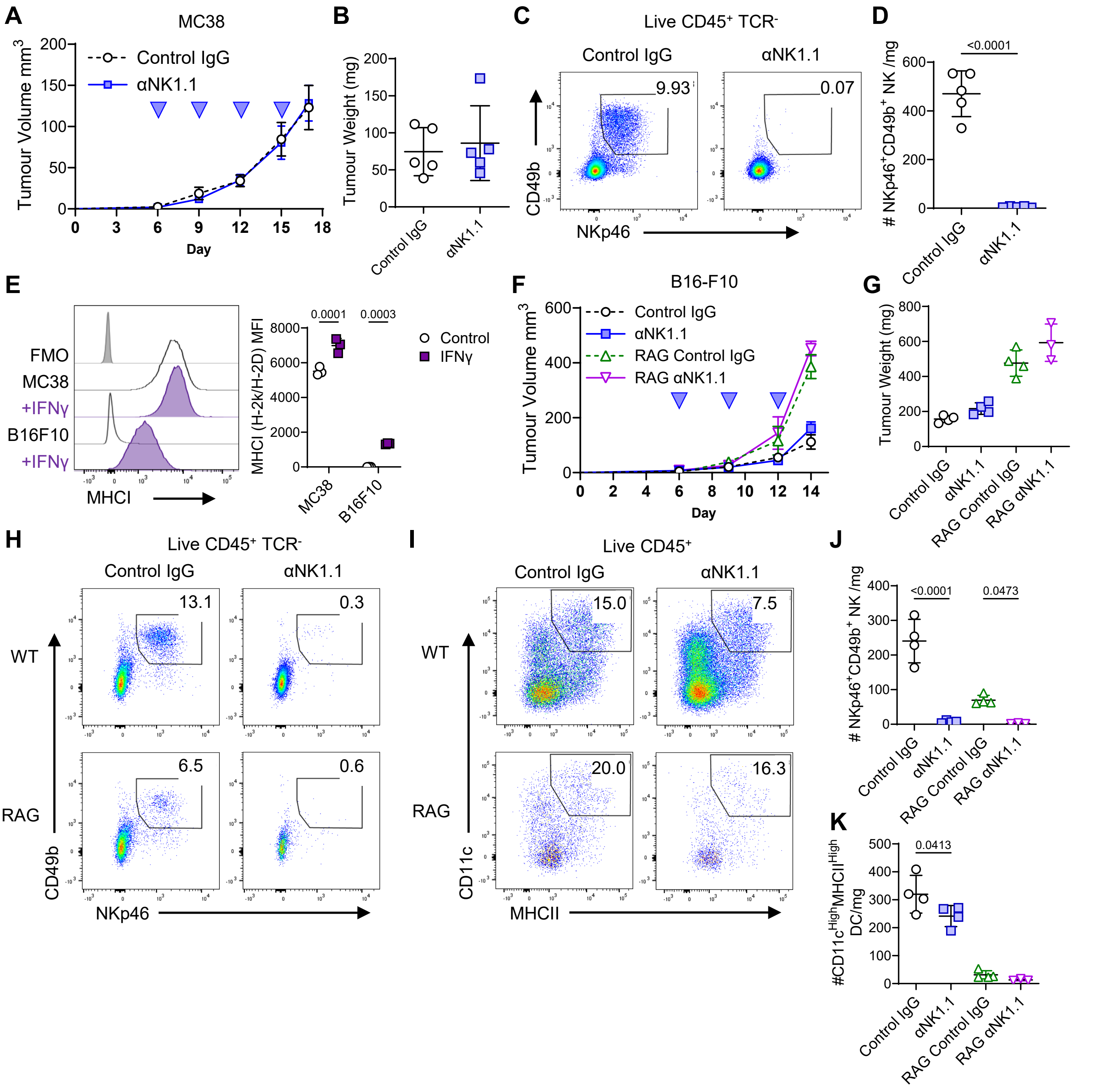

Supplementary Fig. 13. Depletion of NK cells after subcutaneous tumors have become established has minimal impact on tumor growth.

To assess the contribution of NK cells to the control of established tumors, MC38 or B16-F10 tumors were engrafted subcutaneously on the flank of mice and administered with anti-NK1.1 depleting or isotype control antibodies from day 6 of tumor growth. (A) Tumor growth curves of MC38 tumors treated with anti-NK1.1 or IgG control antibodies (indicated by red arrows). (B) Tumor weight upon tissue harvest. (C) Flow cytometry plots showing efficient depletion of NKp46+ CD49b+ cells in the MC38 tumors with anti-NK1.1 antibodies. (D) Enumeration of the number of NK cells present within MC38 tumors. Data from one independent experiment, n=5. (E) Flow cytometry histogram showing MHCI expression on MC38 and B16-F10 cell lines +/- IFNγ, alongside MFI, n=3. (F) Tumor growth curves of B16-F10 tumors grafted into WT or Rag-/- mice and treated with anti-NK1.1 or IgG control antibodies (indicated by red arrows), n=3 for Rag-/- mice treated with anti-NK1.1 and n=4 for all other conditions. (G) Tumor weight upon tissue harvest. (H) Flow cytometry plots showing efficient depletion of NKp46+ CD49b+ cells in the MC38 tumors with anti-NK1.1 antibodies. (I) Flow cytometry plots depicting identification of CD11c+ MHCII+ DC within B16-F10 tumors from WT and RAG2 mice. (J) Enumeration of the number of NK cells present within MC38 tumors. (K) Enumeration of intratumoral DC normalized to B16-F10 tumor weight in each treatment group. Statistical significance was determined by (B, D) unpaired *t*-tests; (E) two-way ANOVA with Šidák's multiple comparisons test; (J, K) one-way ANOVA with Tukey's multiple comparison test comparing means of pre-selected pairs (IgG control to anti-NK1.1, and RAG IgG to RAG anti-NK1.1). Data are presented showing all individual data points as well as the mean value +/- SD throughout, except in (A), where mean +/- SEM are shown.

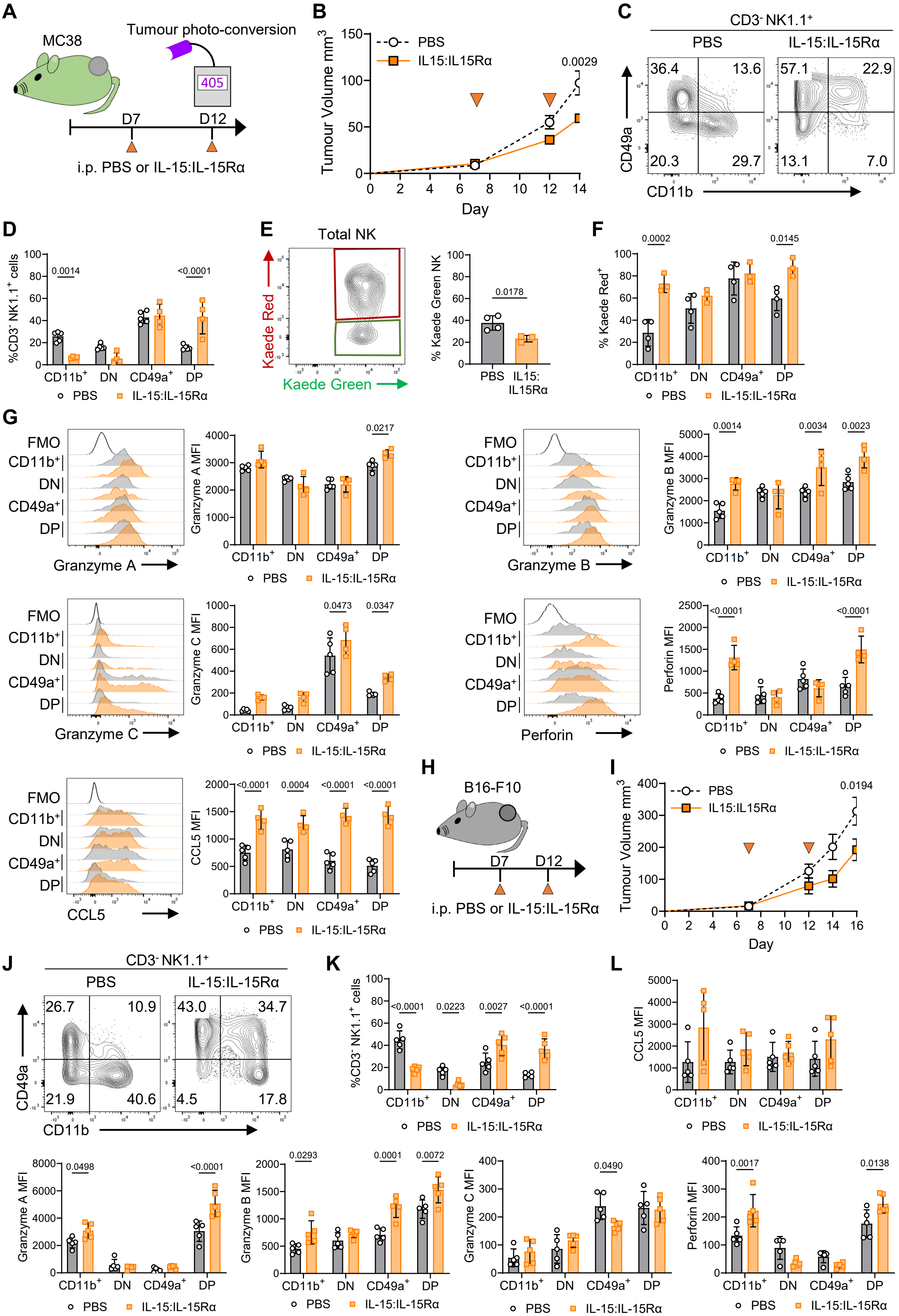

A
MC38
Tumour photo-conversion
405
D7
D12
i.p. PBS or IL-15:IL-15Rα
B
Tumour Volume mm3
PBS
IL15:IL15Rα
0.0029
Day
C
CD3- NK1.1+
PBS
IL-15:IL-15Rα
36.4
13.6
20.3
29.7
57.1
22.9
13.1
7.0
CD49a
CD11b
D
%CD3- NK1.1+ cells
0.0014
<0.0001
CD11b+ DN CD49a+ DP
PBS IL-15:IL-15Rα
E
Total NK
Kaede Red
Kaede Green
% Kaede Green NK
0.0178
PBS IL15: IL15Rα
F
% Kaede Red+
0.0002
0.0145
G
FMO
CD11b+
DN
CD49a+
DP
Granzyme A
Granzyme A MFI
0.0217
Granzyme B
Granzyme B MFI
0.0014
0.0034
0.0023
Granzyme C
Granzyme C MFI
0.0473
0.0347
Perforin
Perforin MFI
<0.0001
<0.0001
CCL5
CCL5 MFI
<0.0001
0.0004
<0.0001
<0.0001
H
B16-F10
D7
D12
i.p. PBS or IL-15:IL-15Rα
I
Tumour Volume mm3
PBS
IL15:IL15Rα
0.0194
Day
J
CD3- NK1.1+
PBS
IL-15:IL-15Rα
26.7
10.9
21.9
40.6
43.0
34.7
4.5
17.8
CD49a
CD11b
K
%CD3- NK1.1+ cells
<0.0001
0.0223
0.0027
<0.0001
L
CCL5 MFI
Granzyme A MFI
0.0498
<0.0001
Granzyme B MFI
0.0293
0.0001
0.0072
Granzyme C MFI
0.0490
Perforin MFI
0.0017
0.0138
CD11b+ DN CD49a+ DP
PBS IL-15:IL-15Rα

Supplementary Fig. 14. Administration of IL-15:IL-15Rα complexes enhances tumor control in MC38 and B16-F10 models.

MC38 tumor cells were engrafted into C57BL/6 Kaede mice and administered with either PBS or IL-15:IL-15Rα complexes on day 7 and 12 of tumor growth. (A) Cartoon diagram showing experimental setup. (B) MC38 tumor growth curve from 1 independent experiment (PBS n=5, IL-15:IL-15Rα n=4). (C) Representative flow cytometry plots showing CD11b and CD49a integrin expression on NK cells (CD3- NK1.1+) isolated from MC38 tumors. (D) Proportion of NK cells with CD11b+CD49a-, CD11b-CD49a-, CD11b-CD49a+, and CD11b+CD49a+ phenotype. (E) Representative flow cytometry plot showing Kaede Green versus Kaede Red expression by NK cells, alongside enumeration of % Kaede Green+ NK cells in either PBS (n=4) or IL-15:IL-15Rα (n=3) treatment. (F) Proportion of Kaede Red+ NK cells across NK cell subsets in either treatment. (G) Representative histograms and enumeration of Granzyme A, Granzyme B, Granzyme C, Perforin, and CCL5 production (MFI) by NK cells from MC38 tumors after *ex vivo* stimulation. (H) Cartoon showing experimental setup whereby B16-F10 tumors were engrafted into WT C57BL/6 mice and administered with i.p. injection of PBS or IL-15:IL-15Rα complexes on day 7 and 12 of tumor growth. (I) B16-F10 tumor growth from 1 independent experiment (PBS n=5, IL-15:IL-15Rα n=5). (J) Representative flow cytometry plots displaying CD11b versus CD49a expression, and (K) enumeration of each NK cell subset comprising intratumoral NK cells. (L) Enumeration of CCL5, Granzyme A, Granzyme B, Granzyme C, and Perforin production (MFI) by NK cells from B16F10 tumors after *ex vivo* stimulation. Statistical significance was determined by (B, D, F, G, I, K, L, M) two-way ANOVA with Šidák's multiple comparisons test; (E) unpaired t-test. Data are presented showing all individual data points as well as the mean value +/- SD throughout, except in (B, I), where mean +/- SEM are shown.
