# Supplementary material for: Rapid functional impairment of natural killer cells following tumor entry limits anti-tumor immunity
Source: Nat Commun. 2024 Jan 24;15:683. doi: 10.1038/s41467-024-44789-z (PMC10808449; doi:10.1038/s41467-024-44789-z)
Supplement: Supplementary file 3 — Reporting Summary [file 41467_2024_44789_MOESM3_ESM.pdf]

nature portfolio

Corresponding author(s): David Withers
Last updated by author(s): 13/12/2023

Reporting Summary

Nature Portfolio wishes to improve the reproducibility of the work that we publish. This form provides structure for consistency and transparency in reporting. For further information on Nature Portfolio policies, see our Editorial Policies and the Editorial Policy Checklist.

Please do not complete any field with "not applicable" or n/a. Refer to the help text for what text to use if an item is not relevant to your study.
For final submission: please carefully check your responses for accuracy; you will not be able to make changes later.

Statistics

For all statistical analyses, confirm that the following items are present in the figure legend, table legend, main text, or Methods section.

n/a	Confirmed	
☐	☒	The exact sample size (*n*) for each experimental group/condition, given as a discrete number and unit of measurement
☐	☒	A statement on whether measurements were taken from distinct samples or whether the same sample was measured repeatedly
☐	☒	The statistical test(s) used AND whether they are one- or two-sided<br>*Only common tests should be described solely by name; describe more complex techniques in the Methods section.*
☒	☐	A description of all covariates tested
☐	☒	A description of any assumptions or corrections, such as tests of normality and adjustment for multiple comparisons
☐	☒	A full description of the statistical parameters including central tendency (e.g. means) or other basic estimates (e.g. regression coefficient) AND variation (e.g. standard deviation) or associated estimates of uncertainty (e.g. confidence intervals)
☐	☒	For null hypothesis testing, the test statistic (e.g. *F*, *t*, *r*) with confidence intervals, effect sizes, degrees of freedom and *P* value noted<br>*Give P values as exact values whenever suitable.*
☒	☐	For Bayesian analysis, information on the choice of priors and Markov chain Monte Carlo settings
☒	☐	For hierarchical and complex designs, identification of the appropriate level for tests and full reporting of outcomes
☒	☐	Estimates of effect sizes (e.g. Cohen's *d*, Pearson's *r*), indicating how they were calculated

Our web collection on statistics for biologists contains articles on many of the points above.

Software and code

Policy information about availability of computer code

Data collection	Flow Cytometric analysis and cell sorting were performed using BD FACSAria II and BD FACSFortessa running BD FACSDiva software Version 8.0.1<br>RNA sequencing: Gene expression libraries were prepared from FACS-sorted cells using the 10X Genomics Chromium Controller. Paired-end sequencing was performed on the Illumina NovaSeq 6000 platform
Data analysis	Flow cytometric analysis were performed using BD FlowJo version 10.8.1 and GraphPad Prism 9.4.0<br>RNA sequencing analysis: Python v3.8.12, R v4.0.4, bclfastq software v2.20.0.422, FastQC v0.11.3, FastqScreen v0.9.2, FastqStrand v0.0.5, CellRanger v6.1.2, Scanpy v1.8.2, Scrublet v0.2.1, UMAP v0.5.1, msigdbr v7.5.1, tradeSeq v0.99, Cibersortx (web tool), fgsea v1.24, pySCENIC v0.12, CellChat v1.5.0, harmonypy v0.0.5

For manuscripts utilizing custom algorithms or software that are central to the research but not yet described in published literature, software must be made available to editors and reviewers. We strongly encourage code deposition in a community repository (e.g. GitHub). See the Nature Portfolio guidelines for submitting code & software for further information.

Data

Policy information about availability of data

All manuscripts must include a data availability statement. This statement should provide the following information, where applicable:
- Accession codes, unique identifiers, or web links for publicly available datasets
- A description of any restrictions on data availability
- For clinical datasets or third party data, please ensure that the statement adheres to our policy

The scRNA-seq data generated by this study have been deposited in the GEO public repository under accession numbers GSE221064 https://www.ncbi.nlm.nih.gov/geo/query/acc.cgi?acc=GSE221064
Previously published scRNA-seq data used in this paper can be accessed under the accession number GSE178341.

Research involving human participants, their data, or biological material

Policy information about studies with human participants or human data. See also policy information about sex, gender (identity/presentation), and sexual orientation and race, ethnicity and racism.

Reporting on sex and gender	The population included two female and eight male patients, age range 57-90 years (median 81.5 years)
Reporting on race, ethnicity, or other socially relevant groupings	Not done
Population characteristics	Paired peripheral blood and non-affected, as well as cancerous gut tissue was obtained from patients undergoing colorectal cancer surgery. The median cancer stage of the population was IVa (range 0-IVb).
Recruitment	Patients undergoing resection surgery for colorectal cancer were informed about the study by the surgeon or research nurse. Written informed consent was collected before surgery.
Ethics oversight	Sample collection was approved by the Swedish Ethical Review Authority.

Note that full information on the approval of the study protocol must also be provided in the manuscript.

Field-specific reporting

Please select the one below that is the best fit for your research. If you are not sure, read the appropriate sections before making your selection.

☒ Life sciences ☐ Behavioural & social sciences ☐ Ecological, evolutionary & environmental sciences

For a reference copy of the document with all sections, see nature.com/documents/nr-reporting-summary-flat.pdf

Life sciences study design

All studies must disclose on these points even when the disclosure is negative.

Sample size	Sample sizes for *in vivo* studies used a minimum of 4 mice per time point/treatment group, and where applicable were repeated to reproducibly observe statistical significance. The number of animals per group was based on previous data published in Li *et al* 2021 JEM. No size calculations were performed for *in vitro* studies, experiments were were repeated (2 independent experiments) and data analysed.
Data exclusions	Two data points were excluded as outliers using the ROUT method from Supplemental figure 14E-F. These tumours were excluded due to incomplete photoconversion caused by their size (being too large).
Replication	All *in vivo* studies, excluding Fig 2D,H, Fig 3G, and Fig 4B,I, were replicated at least twice and observations were reproduced. This was due to other supporting data at different time points negating the necessity to repeat the data, and/or limited availability of mouse strains. In vitro experiments were were replicated twice except for Fig 4I,J which was done once.
Randomization	Cages of mice were randomly designated treatment groups after ensuring tumor size/burden was equal after evaluation by calipers to measure the tumor. For *in vitro* experiments randomization was not performed.
Blinding	Treatment compounds used (i.e anti-NK1.1 or anti-IgG controls) were blinded to the researcher administering them to the mice. That same researcher collected all caliper measurements to ensure consistency within experiments. Analysis of tumor growth and subsequent flow cytometric analyses were not blinded. All other experiments (e.g. in vitro analyses, NK cell characterisation) were not blinded.

Reporting for specific materials, systems and methods

We require information from authors about some types of materials, experimental systems and methods used in many studies. Here, indicate whether each material, system or method listed is relevant to your study. If you are not sure if a list item applies to your research, read the appropriate section before selecting a response.

Materials & experimental systems

n/a	Involved in the study	
☐	☒	Antibodies
☐	☒	Eukaryotic cell lines
☒	☐	Palaeontology and archaeology
☐	☒	Animals and other organisms
☒	☐	Clinical data
☒	☐	Dual use research of concern
☒	☐	Plants

Methods

n/a	Involved in the study	
☒	☐	ChIP-seq
☐	☒	Flow cytometry
☒	☐	MRI-based neuroimaging

Antibodies

Antibodies used

Surface and intracellular antibodies used were against the following mouse antigens: CCL5 purified (1:200, Goat IgG, R&D Systems), CD107a BV786 (1:200, clone 1D4B, BioLegend), CD11b PE-Dazzle594 (1:300, clone M1/70, BioLegend), CD200r1 APC (1:200, clone OX-110, BioLegend), CD3 BV711 or BUV737 (1:200, clone 17A2, BioLegend), CD45 FITC or BV510 (1:200, clone 30-F11, BioLegend), CD49a BUV395 (1:200, clone RM4-5, BD Bioscience), CD49b BV711 (1:100, clone DX5, BioLegend), CD69 PE-Cy7 or BV711 (1:200, clone H1.2F3, BioLegend), DNAM-1 BV605 (1:200, clone TX42.1, BioLegend), EOMES PE or PE-Cy7 (1:100, clone Dan11mag, BioLegend), Granzyme A purified (1:300, clone 3G8.5, BD Biosciences), Granzyme B BV421 (1:300, clone QA18A28, BioLegend), Granzyme C PE-Cy7 (1:300, clone SFC108, BioLegend), IFN BUV737 (1:400, clone XMG1.2, BioLegend), IgG2b BV605 or R718 (1:400, clone R12-3, BioLegend), IL-7R BV605 or BV421 (1:100, clone AFR34A7R34, BioLegend), Ki-67 BV711 or AF700 (1:200, clone SolA15, BioLegend), KLRG1 BV605 (1:100, clone 2F1, BioLegend), LAG3 BV786 (1:100, clone C9B7W, BioLegend), NK1.1 BV650 or BV786 (1:100, clone PK136, BioLegend), NKG2A/C/E BV421 (1:400, clone 20d5, BioLegend), NKp46 BV650 or BV786 (1:100, clone 29A1.4, BioLegend), OX40 BV711 (1:100, clone OX-86, BioLegend), PD-1 PE-Cy7 (1:200, clone RMP1-30, BioLegend), Perforin APC (1:300, clone S16009A, BioLegend), T-bet e660 (1:100, clone eBio4B10, BioLegend), and TCR BUV737 (1:200, clone H57-597, BioLegend).Surface and intracellular antibodies used were against the following human antigens: CD16-FITC (1:25, clone 3G8;Biolegend), NKG2A-APC (3:50, clone Z199, Beckman Coulter), GZA-AF700 (1:50, clone CB9, Biolegend), CD49a-BV421 (1:50, clone SR84;BD), CD11b-BV510 (1:25, clone ICRF44; BD), CD3-BV570 (3:50, clone UCHT1; Biolegend), CD57-BV605 (1:50, clone QA17A04; Biolegend), CD103-BV650 (1:25, clone BER-ACT8; BD), PRF1-BV711 (1:50, clone dG9; Biolegend), LAG3-BV786 (1:25, clone T47-530; BD), T-bet-PE (1:10, clone O4-46;BD), GZB-PE-CF594 (1:25, clone GB11; BD), CD127-PE-Cy5 (3:50, clone A019D5; Biolegend), CD69-PE-Cy5.5 (1:25, clone CH/4; Thermofisher), EOMES-PE-Cy7 (3:50, clone WD1928; Thermofisher), CD45-BUV395 (1:25, clone HI30; BD), CD56-BUV737 (1:25, clone NCAM16.2; BD).

Validation	Validation for all antibodies is available on vendor websites. All Abs were acquired from commercial sources.

Eukaryotic cell lines

Policy information about cell lines and Sex and Gender in Research

Cell line source(s)	All cell lines used and the source are stated under 'Mouse tumor models' within the Materials and Methods section.
Authentication	The cell lines were not authenticated beyond purchasing them from vendoors or acquring them through collaborators
Mycoplasma contamination	All cell lines were checked for Mycoplasma contamination and confirmed as negative
Commonly misidentified lines (See ICLAC register)	No commonly misidentidfied lines were used

Animals and other research organisms

Policy information about studies involving animals; ARRIVE guidelines recommended for reporting animal research, and Sex and Gender in Research

Laboratory animals	C57BL/6 and Rag2$^{-/-}$ mice were obtained from Charles River, and ifng^{mkate2} mice were generated and obtained from Taconic Biosciences. C57BL/6 Kaede mice were obtained from Dr. M. Tomura (Osaka Ohtani University). BALB/c Kaede mice from Dr. Y. Miwa (Tsukuba University) and Dr. O. Kanagawa (RCAI, Riken). Hif1a$^{fl/fl}$NCR1^{iCreTg} were obtained from Dr. C. Stockmann, and were crossed with C57BL/6 Kaede mice within the University of Birmingham Biomedical Services Unit. Mice were maintained and housed at 21°C +/- 2°C, 55% humidity (+/- 10%) with 12 hr light dark/ cycle in 7-7 IVC caging with environmental enrichment at the University of Birmingham Biomedical Services Unit. Mice were used experimentally when aged between 8-18 weeks.
Wild animals	No wild animals were used in this study.
Reporting on sex	Both male and female mice were used within this study.
Field-collected samples	No field samples were collected
Ethics oversight	Animals were used in accordance with Home Office Guidelines under a Project License awarded to D.R. Withers and approved by the University of Birmingham Animal Welfare and Ethical Review Body.

Flow Cytometry

Plots

Confirm that:

- [x] The axis labels state the marker and fluorochrome used (e.g. CD4-FITC).
- [x] The axis scales are clearly visible. Include numbers along axes only for bottom left plot of group (a 'group' is an analysis of identical markers).
- [x] All plots are contour plots with outliers or pseudocolor plots.
- [x] A numerical value for number of cells or percentage (with statistics) is provided.

Methodology

Sample preparation	As described in Materials and Methods 'Tissue dissociation' and 'flow cytometry'.
Instrument	BD FACSFortessa x20
Software	DIVA 8.0.1 and FlowJo version 10.8 were used for acquisition and analysis
Cell population abundance	
Gating strategy	In all flow experiments doublets were excluded using FSC-A/FSC-H, and dead cells excluded using a viability dye. Cells were either gated as CD45+ or Kaede+ depending on the mouse strain used.<br>The strategy used for cell sorting in the scRNA-seq experiment is provided in Supplemental figure 1. In mouse experiments ILCs were gated as NK1.1 before further gating. All subsequent gating that differs is included within each figure.<br>Gating for human tissues is included in Supplementary Figure 12.

- [x] Tick this box to confirm that a figure exemplifying the gating strategy is provided in the Supplementary Information.
