# Supplementary material for: Rapid functional impairment of natural killer cells following tumor entry limits anti-tumor immunity
Source: Nat Commun. 2024 Jan 24;15:683. doi: 10.1038/s41467-024-44789-z (PMC10808449; doi:10.1038/s41467-024-44789-z)
Supplement: Supplementary file 4 — Description of Additional Supplementary Files [file 41467_2024_44789_MOESM4_ESM.pdf]

Description of Additional Supplementary Files

Supplementary Data 1. DEGs between *Itgam*-expressing and *Itga1*-expressing tumor NK cells in murine MC38 tumours and human CRC
